# Time of first contact determines cooperator success in a three-member microbial consortium

**DOI:** 10.1093/ismeco/ycaf004

**Published:** 2025-01-13

**Authors:** Rachel Los, Tobias Fecker, P A M van Touw, Rinke J van Tatenhove-Pel, Timon Idema

**Affiliations:** Department of Bionanoscience, Kavli Institute of Nanoscience, Delft University of Technology, Van der Maasweg 9, 2629 HZ Delft, The Netherlands; Department of Biotechnology, Delft University of Technology, Van der Maasweg 9, 2629 HZ Delft, The Netherlands; Department of Bionanoscience, Kavli Institute of Nanoscience, Delft University of Technology, Van der Maasweg 9, 2629 HZ Delft, The Netherlands; Department of Biotechnology, Delft University of Technology, Van der Maasweg 9, 2629 HZ Delft, The Netherlands; Department of Bionanoscience, Kavli Institute of Nanoscience, Delft University of Technology, Van der Maasweg 9, 2629 HZ Delft, The Netherlands

**Keywords:** cross-feeding, cooperation, population dynamics, spatial organization, individual-based modelling

## Abstract

Microbial communities are characterized by complex interaction, including cooperation and cheating, which have significant ecological and applied implications. However, the factors determining the success of cooperators in the presence of cheaters remain poorly understood. Here, we investigate the dynamics of cooperative interactions in a consortium consisting of a cross-feeding pair and a cheater strain using individual-based simulations and an engineered *L. cremoris* toy consortium. Our simulations reveal first contact time between cooperators as a critical predictor for cooperator success. By manipulating the relative distances between cooperators and cheaters or the background growth rates, influenced by the cost of cooperation, we can modulate this first contact time and influence cooperator success. Our study underscores the importance of cooperators coming into contact with each other on time, which provides a simple and generalizable framework for understanding and designing cooperative interactions in microbial communities. These findings contribute to our understanding of cross-feeding dynamics and offer practical insights for synthetic and biotechnological applications.

## Introduction

Microorganisms often live together in surface-attached communities of many strains and species, called biofilms. An early stage of the planktonic cell to biofilm life cycle is microcolonies, which make up the initial kernels that later grow into larger biofilms [1]. Studying the formation of these microcolonies provides valuable insight into biofilm development [2]. The organisms living in biofilms can form complicated networks of antagonistic, mutualistic, competing or cooperating interactions [3–5]. One of the ways species can cooperate is by cross-feeding, where both species produce metabolites or other essential compounds that benefit the other [6]. The production of these compounds often comes at a cost for the producer in the form of extra expended energy (e.g. in the form of ATP). This creates opportunities for cheater species to exploit cooperators, by reaping the same benefits from the cross-fed compounds without contributing to the cooperation themselves [7]. Studying the interactions in bacterial systems can have implications for applications concerning biofilms in medical, biotechnological, and industrial contexts [8–10]. Moreover, because mutualism and cooperative interactions can be observed in all branches of the phylogenetic tree, there is general interest in what makes this type of social behaviour evolutionary stable [7]. Due to their relative simplicity, microbial systems can be used as model systems for studying broader social behaviours in biology [11]. The interactions between microorganisms are in general complex, with many inter-dependent variables that are difficult to isolate [12]. An important factor on the dynamics of any collection of interacting species is spatial organization [13–18]. This spatial structure plays a role in both the emergence and the maintenance of cooperator co-existence [19]. For example, spatially structured environments can promote clonal patching, which is useful for intra-species cooperation [20, 21] as well as pattern formation in multispecies colonies [16, 22].

In this paper, we employ individual-based modelling (IBM) to simulate a consortium of two cooperating species and a selfish cheater growing on a surface. By incorporating spatial structure and proximity in metabolic interactions, we attempt to uncover the factors that govern cooperative success in the face of cheating [23]. This will contribute to our understanding of population dynamics in cooperative biological systems.

## Materials and methods

### Individual-based model

**Figure 1:**
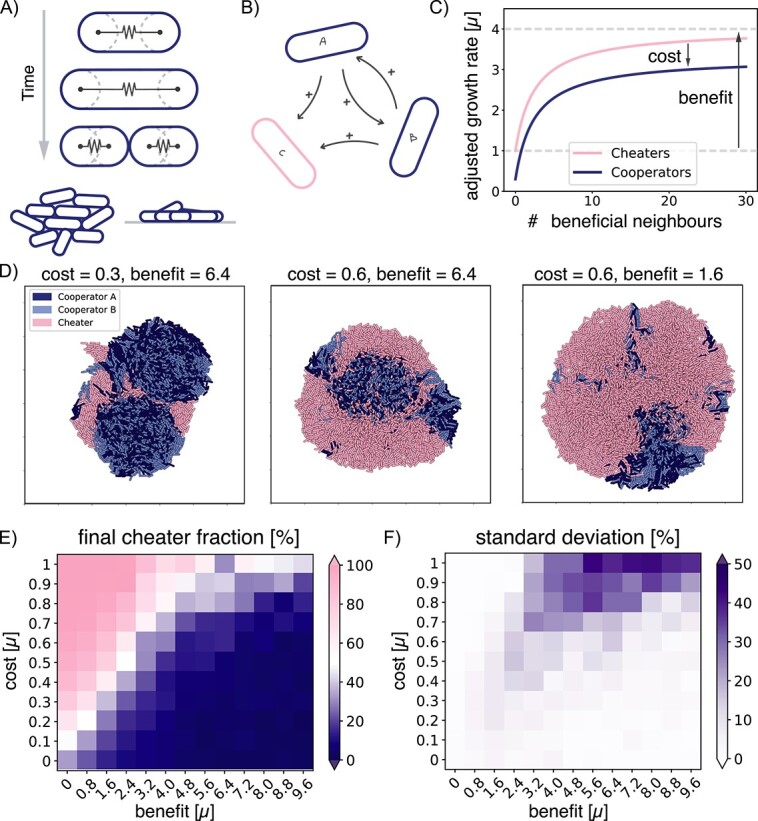
Effect of cost and benefit of cooperation on cooperator success in individual-based simulations. (A) Schematic representation of the individual-based model. Spherocylindrical particles grow and divide, forming a 3D colony. (B) The simulated consortium of two cooperators, A and B, that benefit each other and a cheater C, which benefits from both cooperators A and B but does not reciprocate. (C) Growth rate of both the cooperators and the cheaters, dependent on the number of beneficial neighbours (i.e. neighbours that provide a useful compound). The growth rate is affected by the cost and benefit of cooperation. (D) Snapshots of the final 3D colonies for different combinations of cost and benefit. Area shown is always $130\times 130\,\mu \text{m}^{2}$. (E) Final cheater fractions for varying cost and benefit, with averages taken over 15 runs. (F) The standard deviation of those runs.

In our simulations, we initialize spherocylindrical particles on a surface and let them grow, divide, and interact with each other [24]. The mechanical interactions of the particles are the same for all species: in addition to steric repulsion between the particles, they have an attractive potential, which makes them stick together and to the surface (see Section S1 of the supplementary material). The cross-feeding interaction between the different species is implemented by adjusting their growth rates based on their immediate environment (Fig. 1C) [25, 26]. In particular, for every growth step, we count for each particle how many beneficial neighbours they have in their immediate vicinity and use this number as input for a scaled Monod equation: 


(1) \begin{align*} & \mu^{\prime}_{A} = \mu^{\prime}_{B} = \mu \left(1 + \frac{n}{K_{\text{s}} + n} b - c\right), \end{align*}

(2) \begin{align*} & \mu^{\prime}_{C} = \mu \left(1 + \frac{n}{K_{\text{s}} + n} b\right),\end{align*}

which determines the growth rate of the particle, $\mu ^{\prime}$. Here, $n$ is the number of beneficial neighbours, $K_{\text{s}}$ is the Hill coefficient, which we set to $2.5$ as this is the average number of beneficial direct neighbours in a well-mixed colony of $10^{4}$ particles, and $\mu $ is the background growth of the cheaters (i.e. the growth rate of an isolated cheater particle). Both benefit, $b$, and cost, $c$, are given as fractions of $\mu $, where $b$ sets the maximal growth rate a particle can achieve and $c$ gives a downward shift, which can have any value between $0$ and $1$.

### Cross-feeding simulations

We implement a symmetric cross-feeding interaction by having species A and B increase their growth rate depending on how many of their neighbours are B and A, respectively. C increases its growth rate based on the minimum number of A and B in its vicinity. At the beginning of the simulation, $10$ particles of each species are randomly initialized on a $50\times 50\,\mu \text{m}^{2}$ patch of surface. We grow the system to a final size of $10^{4}$ particles.

### Asymmetric simulations

For the asymmetric case that resembles our experimental consortium, the interactions remain the same except that, for the growth rate of C, $n$ is taken to be the number of B particles in the neighbourhood. Additionally, $100$ particles are initialized in different A:B:C ratios, and the system is grown to a final size of $7\times 10^{3}$ particles.

### Microbial strains and growth conditions

Strains and plasmids used in this study are listed in Table 1. All strains were grown on CDMpc, described by Price *et al.* [27] at $30\,^\circ \text{C}$ in a stationary incubator. We used glucose and lactose as carbon sources, in the concentrations as indicated. Where indicated, the strains were grown on CDMpc_cas_, containing 0.2 wt% casein sodium salt (from bovine milk, #C8654, Sigma Aldrich, Saint Louis, MO, USA) instead of amino acids. To prepare agar plates, liquid medium was supplemented with 2 wt% BD Difco^TM^ Bacto^TM^ Agar (BD, NJ, USA).

**Table 1:** Strains and plasmids used in this study.

	**Description**	**Reference**
**Strains**		
NZ9000 Glc$^{-}$ Lac^+^	*L. cremoris* NZ9000*$\Delta $glk$\Delta $ptnABCD*, carrying pMG820	[28]
MG610	*L. cremoris* MG1363 with two *prtMP* copies integrated into the genome; erythromycin resistant	[29]
MG1363_GFP	*L. cremoris* MG1363 with Dasher-GFP gene integrated into the genome; erythromycin resistant	[30]
MG1363_GFP Lac^+^	MG1363_GFP carrying pMG820; erythromycin resistant	This study
MG5267	*L. cremoris* MG1363 with lactose operon integrated into the genome	[45]
**Plasmids**		
pMG820	23.7 kb lactose miniplasmid containing *lacFEGABCD*	[46]

*L. cremoris* NZ9000 Glc$^{-}$ Lac^+^ [28], *L. cremoris* MG5267 and MG1363_GFP Lac^+^ (this study) were precultured in 25 ml CDMpc + 0.09 wt% lactose, and *L. cremoris* MG610 [29] and MG1363_GFP [30] were precultured in 25 ml CDMpc + 0.09 wt% glucose. Freezer stocks were prepared by growing the strains in CDMpc with the appropriate carbon source and storing the stationary culture at −80$^\circ \text{C}$ with 20 vol% glycerol. Where indicated, erythromycin (Sigma-Aldrich, 856193, Saint Louis, MO, USA) was added at the indicated concentration. For the isolation of pMG820 and transformation into MG1363_GFP, M17 medium was used (from powder, Thermo Fisher Scientific, MA, USA).

### Isolation of pMG820 and transformation into MG1363_GFP

To construct MG1363_GFP Lac^+^, pMG820 was isolated from 5 ml of a stationary NZ9000 Glc$^{-}$ Lac^+^ culture, cultivated in M17 + 0.5 wt% lactose, as follows. The culture was centrifuged using a Sorvall ST40 centrifuge (Thermo Fisher Scientific, MA, USA) (10 min, 5000 g), and resuspended in 30 mM Tris-Hcl pH 8, 3 mM MgCl_2_, 25 wt% sucrose and 2 mg ml$^{-1}$ lysozyme (from egg white, 10837059001 Roche, Basel, Switzerland) and incubated for 30 min at 37$^\circ \text{C}$. Afterwards, the plasmid was isolated using the GeneJET Plasmid Miniprep Kit (Thermo Fisher Scientific, MA, USA). Once obtained, the plasmid was transformed into MG1363_GFP as described by Wells et al. [31] with the following adaptations. The MG1363_ GFP cells were precultured in 50 ml M17 broth with 17 wt% (0.5 M) sucrose, 2.5 wt% glycine and 0.5 wt% glucose at 30$^\circ \text{C}$. After overnight incubation, they were centrifuged (6000 g, 20 min) and washed with 400 ml 17 wt% (0.5 M) sucrose, 10 wt% glycerol (4$^\circ \text{C}$), spun down and resuspended in 200 ml 17 wt% (0.5 M) sucrose, 10 wt% glycerol + 50 mM EDTA (4$^\circ \text{C}$). After incubating on ice for 15 min and spinning down (6000 g, 10 min), the cells were washed again as described above and resuspended in 4 ml 17 wt% (0.5 M) sucrose, 10 wt % glycerol (4$^\circ \text{C}$). Then, 40 ${\mu }$l of the cell solution with 1 ${\mu }$l DNA (100 ng ${\mu }$l$^{-1}$ in Tris-Buffer) was added to a chilled cuvette and pulsed using a Bio-rad Genepulser (Bio-Rad, CA, USA) for 5.7 ms (2000 V, 25 ${\mu }$F, 200$\Omega $), after which 1 ml M17 broth with 17 wt% (0.5 M) sucrose, 2.5 wt% glycine, 0.5 wt% glucose, 20 mM MgCl_2_ and 2 mM CaCl_2_ was added. The solution was added to M17 agar plates containing 0.5 wt% lactose. After incubation for 48 h, colonies were picked and restreaked.

### Growth rate determination

To check the phenotype of *L. cremoris* MG1363_GFP Lac^+^, we tested whether the strain was fluorescent, and whether the growth rate was similar to the growth rate on lactose of *L. cremoris* MG5267, a lactose-positive *L. cremoris* strain, and similar on glucose to its ancestor strain MG1363. Fluorescence of the strain was confirmed using flow cytometry (Accuri C6, BD, NJ, USA). To prepare the cells for inoculation for the growth rate determination, MG1363_GFP Lac^+^ was precultured in CDMpc on both glucose and lactose, and MG5267 and MG1363 were precultured as described above. After 16 h, the OD_660_ was determined using a Jenway 7200 Spectrophotometer (Cole-Palmer, Stone, UK) and the cells were inoculated in duplicates in 30 ml of the same medium as in the preculture at an OD_660_ of 0.01. To determine the growth rate, the OD_660_ was measured every hour (Fig. S5).

### Co-cultivation on plates

*L. cremoris* MG1363_GFP Lac^+^, NZ9000 Glc$^{-}$ Lac^+^ and MG610 were precultured as described above, spun down (8000 g, 15 min), washed in sterile PBS and resuspended in PBS to a final concentration of $3\times 10^7$ cells ml$^{-1}$, as determined by flow cytometry (Accuri C6, BD, NJ, USA). To make the cell mixtures, cells were added together to the indicated cell concentration and strain ratio. Of the cell mixtures, 100 ${\mu }$l was added to plates containing CDMpc_cas_.The plates were incubated for 90 h at 30$^\circ \text{C}$. Afterwards, the cells were removed from the plate by adding 2 ml sterile PBS and spreading it using a sterile spreader, as described in [32]. The cell suspension was removed from the plate, and the cell concentration and cheater abundance was determined using flow cytometry (Accuri C6, BD, NJ, USA). The final cell concentration was then used to calculate a total final cell count. Together with the known initial cell count, this was used to calculate the amount of times the cells had doubled using the following formula: 


(3) \begin{align*}& d = \text{log}_{2} \left( \frac{N_{f}}{N_{i}}\right).\end{align*}

Here, $d$ is the amount of doublings, and $N_{f}$ and $N_{i}$ are the final and initial cell counts, respectively. To selectively lower the growth rate of cooperator A, erythromycin was added to the agar plates at the indicated concentrations.

## Results and discussion

### Cooperator success is highly dependent on initial placement of particles

To explore the impact of local cross-feeding interactions on the development of a multi-species microcolony, we modelled interacting particles growing on a surface (Fig. 1A). We defined three different species: two cooperators A and B, and a cheater C (Fig. 1B). We made use of the fact that metabolic interactions have been shown to take place at very short distances and have the particles adjust their growth rate based on the number of their beneficial nearest neighbours [25, 26]. The strength of the cross-feeding interaction between cooperators A and B is characterized by two main factors: the metabolic cost the cooperators pay to contribute to the cooperation, and the added growth benefit they experience from this interaction. The cheaters do not pay the cost but experience the same benefit (Fig. 1C). For now, the interaction with the cooperators is symmetric, in the sense that the cheater responds similarly to cooperator A as to cooperator B and it needs both to grow. Later in this paper, we also explore asymmetric cheater interactions. The particles are spherocylindrical particles that grow and divide. We start each run with 10 of each species randomly distributed on a surface. We then let them grow until the colonies reach a size of $10^{4}$ particles.

In order to understand how the strength of the local interactions shaped the development of the microcolony, we simulated growing colonies for varying costs and benefits, and recorded the cheater fraction. Typical simulation snapshots of the final colonies are shown in Fig. 1D. The final cheater fractions averaged over 15 runs are shown in Fig. 1E. The bottom-left data point represents a situation with no cost and no benefit, which means that the particles are not interacting at all and their growth rate remains the same. As the cost of cooperation increases while the benefit stays zero, the final cheater fraction increases. In turn, increasing the benefit decreases the cheater fraction and favours the cooperators. Interestingly, there are not many intermediate values for the final cheater fraction, showing more switch-like behaviour than would be expected from a well-mixed system (Fig. S2).

Surprisingly, the simulations gave a large variation between runs with the same interaction parameters, particularly for higher costs and benefits (Fig. 1F). Here, we found standard deviations of up to 50%, which indicates that the final cheater fraction can be anywhere between 0% and 100% for simulations with the same interaction input parameters. Because the only difference between these runs was the initialization of the 30 particles at the start of the run, the large variance had to be a result of the initial placement of the particles, which we explore next.

### First contact time determines cooperator success

In order to investigate how the initial placement of the particles leads to different final cheater fractions, we visually inspected the resulting colonies of the simulations. In general, we observed that colonies were fairly segregated, akin to clonal patches observed in 2D colonies [9]. The cooperator patches are often well mixed, with equal amounts of A and B, which we expect is due to a combination of nematic mixing and the mutualism between these species [20, 33, 34]. In different simulations with the same input parameters, we observed a varying number of these patches of mixed cooperators (Fig. S1). Because the cooperators depend on each other’s proximity to cross-feed, we speculated that the final cheater fractions might be determined by how likely it is for cooperators to meet.

**Figure 2:**
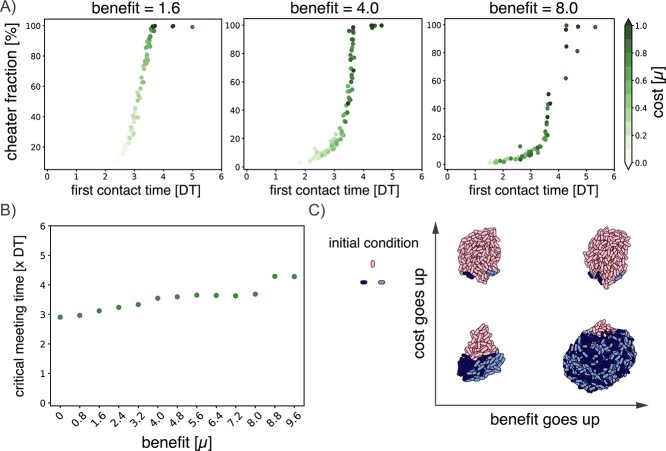
First contact time determines cooperator success. (A) Final cheater fraction of single runs plotted as a function of first encounter time (measured in DT $ =10^{5}$ simulation time steps) for three different benefits. Cost is denoted in green. (B) Critical meeting time as a function of benefit. (C) Toy system for a well-defined initial condition of three particles positioned in a triangle and the resulting colonies, for the combinations of two values for the cost ($0.2$ and $0.7$) and two values for the benefit ($5.6$ and $8.8$).

In order to investigate this relation between the likelihood of meeting and final cheater fraction, we took all the runs and we recorded the first time point where a cooperator A particle comes into contact with a cooperator B particle. Plotting the final cheater fraction as a function of these first contact times for each value of the benefit yielded a clearly defined curve with comparatively little variance (Fig. 2A). Therefore, for a given benefit, the time of first contact was a more informative predictor for cooperator success than the cost of cooperation.

This dependence on cooperators encountering each other is in line with earlier findings on the importance of co-localization probabilities in a similar consortium growing in microdroplets [35]. Other work on two-species cooperator–defector consortia growing plates showed the importance of founder cell configuration [36]. Additionally, expansion–collision dynamics and initial distance between microorganisms have been shown to be important factors for cooperator–defector co-existence [37]. Although all these systems are not identical, they do point to a general principle in cooperative dynamics.

To explore this further, for each value of the benefit, we determined a critical meeting time before which A and B need to make contact in order to outcompete the cheaters and make up >50% of the final population (Fig. 2B). If the first contact time occurred after this point, cheaters dominated the final colony of $10^{4}$ particles. The critical meeting time depended on the cooperator benefit, where a higher benefit allowed for a later critical meeting time. In this case, once first contact had been made, cooperators were able to make up for lost time by growing faster once they were together. Conversely, if the cooperator benefit was lower, the cooperators needed to meet earlier to outcompete the cheaters.

It is important to note that first contact time is determined not only by the cost and benefit, but also the relative distance between the cooperators in the initial placement. To illustrate this, we visualized the outcome of simulations with a well-defined initial placement of the three particles (Fig. 2C). Here, the initial distance between the particles is always the same, and out of range from each other. The cost then affects the time it takes for the particles to traverse that distance, as the cost affects the growth rate of the particles when they are on their own (i.e. their background growth). A higher cost causes the cooperators A and B to have a lower background growth rate, so they are slower to reach each other. If they are too slow, as shown in the top-left, cheater particles grow in between them before they get the chance to make contact. In this case, having a higher benefit (top-right) will not help the cooperators as they never get to reap the rewards of their cooperation. If the cost is lower and A and B find each other before the critical meeting time (bottom-left), they can compete effectively with the cheaters. When the benefit becomes larger (bottom-right), they can significantly outcompete the cheaters once they meet. In our system of random initial distances between particles, this interplay between distance and background growth is what leads to the high variation in competitive outcome. From these simulation results, we propose that it is not just the initial distances between cooperators or just the details of the cooperative interaction, but it is the first contact time that determines cooperator success in surface-attached colonies.

### Experimental consortium of engineered *L. cremoris*

**Figure 3:**
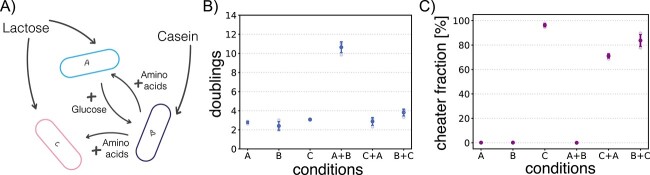
Engineered consortium on agar plates. (A) *L. cremoris* consortium, consisting of cooperators A and B and cheater C. (B) Control experiments showing the amount of doublings for single strain plates and pairwise combinations after three days incubation. (C) Control experiments showing cheater fractions for all single strain plates and pairwise combinations. All conditions are measured in triplicate.

To test if the first contact time between cooperator species is also critical in a biological system, we attempted to translate our simulated consortium to a laboratory setting. We constructed a consortium of three *L. cremoris* strains (Fig. 3A, Table 1) growing on agar plates containing lactose as a carbon source and casein as an amino acid source. Cooperator A, NZ9000 Lac$^{+}$ Glc$^{-}$, can metabolize the galactose moiety of lactose, but exports the glucose moiety from the cell. The glucose can then be used by cooperator B, MG610, which cannot metabolize lactose. In return, MG610 expresses an extracellular enzyme to break down the casein into amino acids; this benefits A, which cannot metabolize the casein. Lastly, we engineered a cheater C, MG1363_GFP Lac$^{+}$, by transforming a plasmid containing the genes necessary to metabolize lactose into MG1363_GFP. This constructed cheater can metabolize lactose and both resulting moieties, but, similar to A, lacks the enzyme to degrade casein. C also expresses GFP, which we use to determine the cheater fraction.

We grew A, B, and C individually on agar plates, and measured the total growth by comparing the final cell count to the initial cell count and calculating the amount of times the cells had doubled. On the single strain plates, there was very little growth, which is what we expected from the designed cross-feeding interaction (Fig. 3B). When growing all the pairwise combinations, we observed that A+B massively outgrew the other combinations. From this, we concluded that, as expected, these two strains experience a strong mutual benefit from growing together. Similarly, we concluded that on plates with C+A and B+C, there is no such mutualism, as the total cell abundance, measured in amount of doublings, stalled. Furthermore, when we measured cheater fractions for these combined plates, we saw that C outgrows both A and B in a pairwise combination, pointing to a general advantage in growth rate that C has over both A and B (Fig. 3C). All in all, we concluded that our experimental consortium accurately represents a cross-feeding pair together with a cheater.

### Relative average distance between cooperators determines cooperator success in plate experiments

To investigate how the first contact time would affect our experimental system, we needed a way to modulate the distance between cooperators on the plates. Unfortunately, it is not so simple to closely control the initial placement or the respective growth rates of the microorganisms on plates. Therefore, we needed a different way to adjust the chance of cooperators meeting each other. Unlike our simulated cheater, the cheater in our experimental consortium is not symmetric in the sense that it does not benefit as much from cooperator A as it does from cooperator B. Because both cooperator A and the cheater C need cooperator B for their amino acid production, we argue that the relative difference between the distance between B and A ($r_{\text{AB}}$) and the distance between B and C ($r_{\text{CB}}$) sets the chance of cooperators meeting. In essence, it is a race between A and C to reach B first.

**Figure 4:**
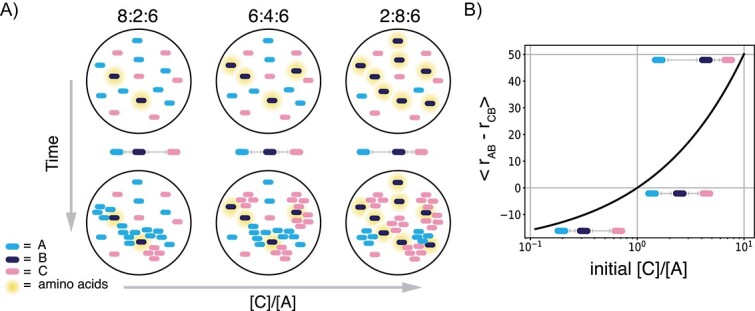
[C]/[A] ratio as a proxy for relative cooperator–cheater distances. (A) Schematic diagram showing how changing the A:B:C ratios is expected to affect the first contact time between cooperators A and B. (B) Relation between the expected average difference between the distance from B to A ($r_{\text{AB}}$) and from B to C ($r_{\text{CB}}$) to the [C]/[A] ratio. For the derivation, see Section S1 of the supplementary material.

To modulate the relative distances between A and B, and between C and B, we adjusted the ratio of [C]/[A]. When [C]/[A] is small ($<1$), there is more A on the plate than C. In this case, the chance of B finding itself close to an A is larger than B finding itself close to a C. If [C]/[A] is large ($>1$), the reverse is true, so on average it is more likely for a B to be surrounded by cheaters (Fig. 4A). The relationship between [C]/[A] and the expectation value of the relative distance can be derived analytically (see Section S1 of the supplementary material and Fig. 4B).

**Figure 5:**
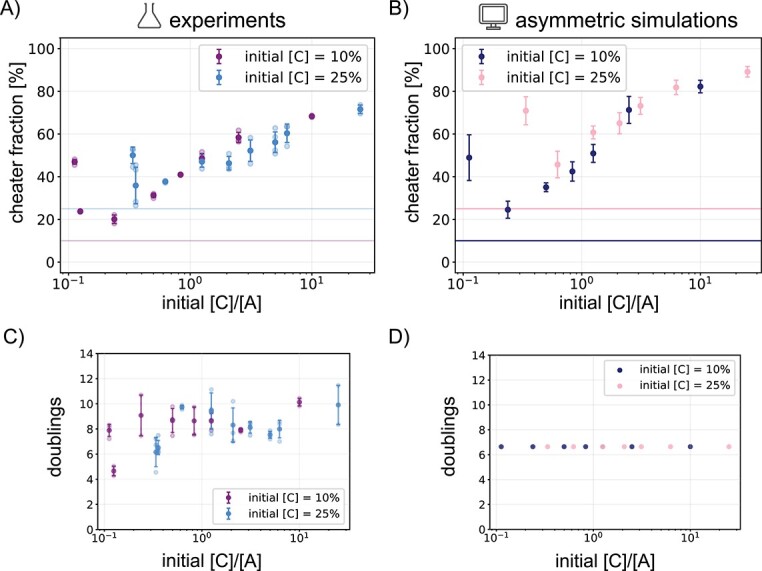
Final cheater fractions depend on initial A:B:C ratios in experiments and simulations. (A) Final cheater fraction of plate experiments with different initial starting ratios of A:B:C, with a constant starting fraction of 10% C in purple and 25% C in blue. Error bars are standard deviation for three replicates. (B) Final cheater fraction for simulations with the same starting ratios of A:B:C. Simulations are of an asymmetric interaction where A and B interact as before, but C only benefits from B. The cost and benefit for these runs are 0.5 and 2.4, respectively. Amount of times the cells doubled between the time of initialization and measurement (C) on the plates and (D) in the simulations.

From our simulation results, we expected the final cheater fraction to rise with a higher relative distance between cooperators, as it would take longer for them to find each other. To test if this would also occur in our experimental consortium, we inoculated cells on plates in different A:B:C ratios, and after three days of incubation we washed the plates and measured the total cell count and the final cheater fraction (Fig. 5A and B). Consistent with our expectations, a small [C]/[A] resulted in final cheater fractions of as low as 20%,where when the ratio was high, the final cheater fraction increased to almost 70%. Note that the total amount of cells after incubation was similar for all starting ratios (Fig. 5C and D).

We show two sets of experiments, one where the initial cheater fraction is always 10% and one where it is 25%. Apart from the leftmost points of the respective curves (discussed below), they fall on to the same curve. From this, we concluded that, regardless of initial cheater fraction, the relative abundance of A and C and therefore the relative average distance between cooperators, determines cooperator success or failure in our experimental consortium.

### Too few nucleation sites result in higher cheater fractions

As shown in Fig. 5A, most data points fall on to the same curve. However, for the smallest [C]/[A] for both the 10% and the 25% curves, the final cheater fractions were higher than expected. These points correspond to A:B:C ratios of 89:1:10 and 74:1:25, respectively, so in both cases there is a minimal amount of cooperator B present. Because there is so much more cooperator A than cheater C, present, we had expected that B coming into contact with A would be inevitable, and therefore the final cheater fraction would be low. Instead, we measured the final cheater fraction at low B to be around 50%.

In order to generate a hypothesis of what happens for low amounts of B, we went back to our simulations. To better reflect the asymmetric interaction the cheater has with cooperators A and B in our experimental consortium, we adapted the model so that the cheater would only benefit from cooperator B, while keeping the interaction between cooperators A and B the same. When we performed these asymmetric simulations for all the ratios we tested in the experiments, the simulations results were similar to the experimental data (Fig. 5A and B). Additionally, we found the same sudden increase in cheater fraction for very low B. Following the trajectories of the composition of these colonies over time, we see that, indeed, B always finds A (Fig. S3). However, there is only one nucleation site, defined as a site where an A and a B are initialized close enough to interact and start a cooperator patch. Because there is only one nucleation site, the development of the colony takes longer, and by the time cooperators A and B have grown to a significant size cooperator patch, the cheaters have already taken up a large part of the colony. This suggests that, next to meeting on time, there is also a minimal amount of nucleation sites necessary for cooperator success.

### Lowering the background growth of cooperators results in higher cheater fractions

**Figure 6:**
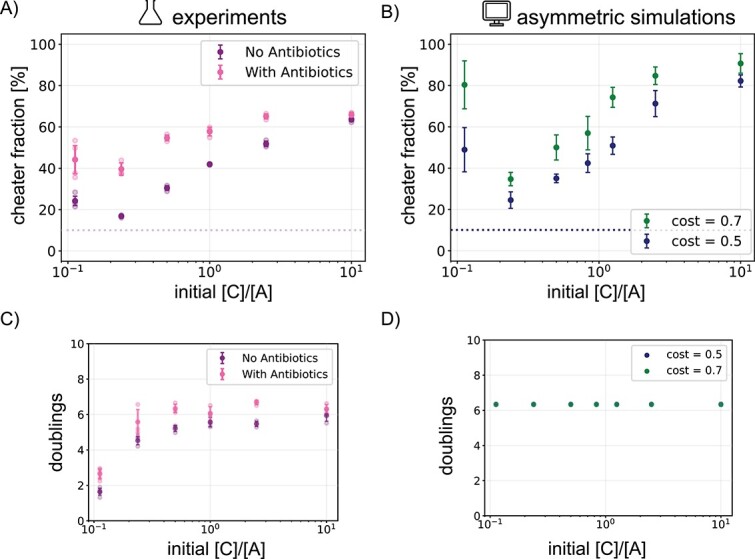
Higher cost of cooperation leads to higher cheater fractions. (A) Final cheater fraction for experiments with different [C]/[A] initial ratios on plates containing $0.04\,\mu \text{g}\,\text{ml}^{-1}$ erythromycin (in pink) and plates without erythromycin (in purple). (B) Final cheater fraction for asymmetric simulations of different [C]/[A] initial ratios for two values of the cost. The benefit is $2.4$ and all data points are averages and standard deviations of five runs. Amount of times the cells doubled between the time of initialization and measurement (C) on the plates and (D) in the simulations.

The symmetric simulations demonstrated that not only the initial distance between cooperators determined cooperator success, but also how fast this distance could be traversed (Fig. 2C). This speed is set by the background growth of the particles. In the simulation, we can set the background growth of the cooperator particles by changing the cost of cooperation (Fig. 2C). A higher cost for cooperators means a lower background growth, which we expected to result in a shift of the curve to the left, where more of cooperator A (i.e. a smaller [C]/[A] ratio) would be needed to achieve the same final cheater fraction. Indeed, increasing the cost in the asymmetric simulations slightly results in a shift to the left, shown as the blue data points in Fig. 6B.

Again, we attempted to test these results from the simulations experimentally. While the cost and benefit of cooperation could not be directly altered, we hypothesized that by selectively inhibiting cooperator A we could achieve a similar effect. To that end, we used erythromycin, which is an antibiotic that only affects the growth of A as both B and C are resistant. We tested for a concentration of erythromycin that would only slightly inhibit the growth of A, which we found to be $0.04\,\mu \text{g}\,\text{ml}^{-1}$ (Fig. S4). We then performed the same experiment for the 10% initial cheater fraction on both normal (untreated) plates and on plates containing the antibiotic (Fig. 6A). For the final cheater fraction on the antibiotic plates, we observed a shift to the left compared with the normal plates, where the same final cheater fraction on standard plates could be achieved on antibiotic plates with a lower ratio of [C]/[A].

Note that in the simulations, we increased the cost for both A and B where, in the experiments, the antibiotic only affects cooperator A. Regardless, we achieved good agreement between the experimental and simulated data. This demonstrates that increasing the cost of cooperation or decreasing the background growth of the cooperators results in higher cheater fractions. Together with our previous results, we propose that this is because the background growth sets the first contact time for the cooperators, which is the main determinant for cooperator success.

The general concept of first contact times being instrumental in cooperator success suggests several strategies for cooperators to increase their survival rate. For instance, cooperators could increase their affinity for each other in solution, forming mixed aggregates. These would then function as the kernels that establish colonies elsewhere, which would greatly improve their chance of survival [1]. Interestingly, the spontaneous developing of cell–cell affinity has been previously shown in mutualistic strains of *E. coli* [16]. Alternatively, chemotactic motility would be a way for cooperators that are already on a surface to establish contact early on, thereby ensuring a beneficial cooperative environment.

Another strategy would be for cooperators to economize on the production of the cross-fed compound when growing on their own, and using quorum sensing to only start production when encountering other cells [38, 39]. Both these strategies could also be explored in synthetic systems making use of specific adhesins and quorum sensing pathways [40, 41].

Not only can the first contact time be exploited by cooperators, it can also provide tactics in a biotechnological application. An example of a widely applied cross-feeding interaction is the yoghurt consortium. Here, protease negative strains can form, which act as a cheater, being able to take over the protease positive strain. Our framework explains why this is less of a problem in a surface-attached or otherwise spatially structured environment [42]. In kombucha production, aggregation between interacting species has a positive effect on the yield [43, 44]. We can now hypothesize that this is due to the metabolically interacting species being in contact early on in the process, outcompeting other strains present and increasing overall yield. Although this should be further tested and verified, the framework proposed in this work should be applicable to similar consortia of different species.

Finally, what this work also shows is that approximating metabolic interactions by only considering the nearest neighbours is a valid way of modelling cross-feeding. Even though our model is quite simple, it is able to capture and predict the behaviour of a real system that is much more complex and has many more interdependent variables than we include in the model. For example, the species in our simulations only differ from each other in how they respond to their neighbours, whereas the strains in our experiment also show variability in overall growth rate. Moreover, in our experiments the nutrient density probably changes over time as the plates grow more dense. Regardless, our simple model of metabolic interactions on a surface is capable of capturing important features of the real system, providing insight into cross-feeding in the presence of cheating.

### Summary

Microbial collaboration is an abundant phenomenon with ecological and applied relevance, yet the factors contributing to cooperator success in the presence of cheaters are poorly understood. We set out to investigate the factors contributing to cooperator success in the presence of an exploiting cheater growing together on a surface. Individual-based simulations of a consortium consisting of three species—a cross-feeding pair and a cheater—indicated a strong influence of the initial placement of the microorganisms on the final outcome. Focusing on the mechanisms, we demonstrated that first contact time was a better predictor for cooperator success than the value of the interaction parameters alone. We then showed how a combination of cost and initial placement together affect this first contact time.

We translated our simulations to an engineered *L. cremoris* toy consortium, consisting of two mutualistic strains and a cheater strain growing on agar plates. We show that by changing the relative distance between cooperators and cheaters by altering the starting ratios of A, B, and C, we could directly influence cooperator success. We recreated our experimental findings by a simple adjustment to the model, making the cheater an asymmetric cheater, further showing how the average distance between cooperators is responsible for cooperator success.

Finally, we showed that, next to the relative distance between cooperators, the time it takes to traverse that distance affects the final cheater fraction as well. This time is set by the background growth, which is dependent on the cost of cooperation in the simulations. In the experiments, we used antibiotics to selectively inhibit the background growth of cooperator A, giving good agreement with the simulations.

We conclude that in a cross-feeding cooperative interaction between strain A and strain B, the ability to find each other on time is the determining factor in cooperator success in the presence of a cheater strain.

Additionally, we have shown that metabolic cross-feeding can be modelled by adjusting the growth rate of particles depending on their nearest-neighbours. Our findings provide better understanding of cross-feeding dynamics in surface-attached communities, as well as an intuitive framework for designing and altering cross-feeding consortia in synthetic and biotechnological applications.

## Supplementary Material

SupplementsRevised_ycaf004

## Data Availability

All experimental data are provided in the article and the online supplementary material. The simulation data can be found on the 4TU repository, at https://doi.org/10.4121/1d711dde-3128-4b14-9a18-444e195361d6.v2. The C++ code used for the simulations can be found at https://gitlab.tudelft.nl/idema-group/costly-cooperation-in-a-microcolony.

## References

[1] Sauer K, Stoodley P, Goeres DM et al. The biofilm life cycle: expanding the conceptual model of biofilm formation. *Nat Rev Microbiol* 2022;20:608–20. 10.1038/s41579-022-00767-0 35922483 PMC9841534

[2] Hall-Stoodley L, Costerton JW, Stoodley P. Bacterial biofilms: from the natural environment to infectious diseases. *Nat Rev Microbiol* 2004;2:95–108. 10.1038/nrmicro821 15040259

[3] Nadell CD, Xavier JB, Foster KR. The sociobiology of biofilms. *FEMS Microbiol Rev* 2009;33:206–24. 10.1111/j.1574-6976.2008.00150.x 19067751

[4] Mitri S, Xavier JB, Foster KR. Social evolution in multispecies biofilms. *Proc Natl Acad Sci* 2011;108:10839–46. 10.1073/pnas.1100292108 21690380 PMC3131810

[5] Deutschmann IM, Lima-Mendez G, Krabberød AK et al. Disentangling environmental effects in microbial association networks. *Microbiome* 2021;9:232. 10.1186/s40168-021-01141-7 34823593 PMC8620190

[6] D’Souza G, Shitut S, Preussger D et al. Ecology and evolution of metabolic cross-feeding interactions in bacteria. *Nat Prod Rep* 2018;35:455–88. 10.1039/C8NP00009C 29799048

[7] West SA, Cooper GA, Ghoul MB et al. Ten recent insights for our understanding of cooperation. *Nat Ecol Evol* 2021;5:419–30. 10.1038/s41559-020-01384-x 33510431 PMC7612052

[8] Conlin PL, Chandler JR, Kerr B. Games of life and death: antibiotic resistance and production through the lens of evolutionary game theory. *Curr Opin Microbiol* 2014;21:35–44. 10.1016/j.mib.2014.09.004 25271120

[9] Frost I, Smith WPJ, Mitri S et al. Cooperation, competition and antibiotic resistance in bacterial colonies. *ISME J* 2018;12:1582–93. 10.1038/s41396-018-0090-4 29563570 PMC5955900

[10] Dieltjens L, Appermans K, Lissens M et al. Inhibiting bacterial cooperation is an evolutionarily robust anti-biofilm strategy. *Nat Commun* 2020;11:107. 10.1038/s41467-019-13660-x 31919364 PMC6952394

[11] Pessione E . The less expensive choice: bacterial strategies to achieve successful and sustainable reciprocal interactions. *Front Microbiol* 2021;11. 10.3389/fmicb.2020.571417 PMC7873842 33584557

[12] Pacheco AR, Segrè D. A multidimensional perspective on microbial interactions. *FEMS Microbiol Lett* 2019;366:fnz125. 10.1093/femsle/fnz125 31187139 PMC6610204

[13] Thébault E, Fontaine C. Stability of ecological communities and the architecture of mutualistic and trophic networks. *Science* 2010;329:853–6. 10.1126/science.1188321 20705861

[14] Van Dyken JD, Müller M, Mack K et al. Spatial population expansion promotes the evolution of cooperation in an experimental prisoner’s dilemma. *Curr Biol* 2013;23:919–23. 10.1016/j.cub.2013.04.026 23664975 PMC4405629

[15] Nadell CD, Drescher K, Foster KR. Spatial structure, cooperation and competition in biofilms. *Nat Rev Microbiol* 2016;14:589–600. 10.1038/nrmicro.2016.84 27452230

[16] Marchal M, Goldschmidt F, Derksen-Müller SN et al. A passive mutualistic interaction promotes the evolution of spatial structure within microbial populations. *BMC Evol Biol* 2017;17:106. 10.1186/s12862-017-0950-y 28438135 PMC5402672

[17] Gupta S, Ross TD, Gomez MM et al. Investigating the dynamics of microbial consortia in spatially structured environments. *Nat Commun* 2020;11:2418. 10.1038/s41467-020-16200-0 32415107 PMC7228966

[18] Gude S, Pinçe E, Taute KM et al. Bacterial coexistence driven by motility and spatial competition. *Nature* 2020;578:588–92. 10.1038/s41586-020-2033-2 32076271

[19] Nadell CD, Foster KR, Xavier JB. Emergence of spatial structure in cell groups and the evolution of cooperation. *PLoS Comput Biol* 2010;6:e1000716. 10.1371/journal.pcbi.1000716 20333237 PMC2841614

[20] Schwarzendahl FJ, Beller DA. Do active nematic self-mixing dynamics help growing bacterial colonies to maintain local genetic diversity? *Front Phys* 2022;10:940980. 10.3389/fphy.2022.940980

[21] Bingham A, Sur A, Shaw LB et al. The effect of cooperator recognition on competition among clones in spatially structured microbial communities. *PLoS One* 2024;19:e0299546. 10.1371/journal.pone.0299546 38547104 PMC10977701

[22] Kayser J, Schreck CF, Yu Q et al. Emergence of evolutionary driving forces in pattern-forming microbial populations. *Phil Trans R Soc B: Biol Sci* 2018;373:20170106. 10.1098/rstb.2017.0106 PMC5904294 29632260

[23] van den Berg NI, Machado D, Santos S et al. Ecological modelling approaches for predicting emergent properties in microbial communities. *Nat Ecol Evol* 2022;6:855–65. 10.1038/s41559-022-01746-7 35577982 PMC7613029

[24] Storck T, Picioreanu C, Virdis B et al. Variable cell morphology approach for individual-based modeling of microbial communities. *Biophys J* 2014;106:2037–48. 10.1016/j.bpj.2014.03.015 24806936 PMC4017289

[25] Dal Co A, van Vliet S, Kiviet DJ et al. Short-range interactions govern the dynamics and functions of microbial communities. *Nat Ecol Evol* 2020;4:366–75. 10.1038/s41559-019-1080-2 32042125

[26] van Tatenhove-Pel RJ, Rijavec T, Lapanje A et al. Microbial competition reduces metabolic interaction distances to the low $\mu $m-range. *ISME J* 2021;15:688–701. 10.1038/s41396-020-00806-9 33077887 PMC8027890

[27] Price CE, Branco dos Santos F, Hesseling A et al. Adaption to glucose limitation is modulated by the pleotropic regulator CcpA, independent of selection pressure strength. *BMC Evol Biol* 2019;19:15. 10.1186/s12862-018-1331-x 30630406 PMC6327505

[28] Pool WA, Neves AR, Kok J et al. Natural sweetening of food products by engineering *Lactococcus lactis* for glucose production. *Metab Eng* 2006;8:456–64. 10.1016/j.ymben.2006.05.003 16844396

[29] Leenhouts KJ, Gietema J, Kok J et al. Chromosomal stabilization of the proteinase genes in Lactococcus lactis. *Appl Environ Microbiol* 1991;57:2568–75. 10.1128/aem.57.9.2568-2575.1991 1768129 PMC183621

[30] van Tatenhove-Pel RJ, Zwering E, Solopova A et al. Ampicillin-treated Lactococcus lactis MG1363 populations contain persisters as well as viable but non-culturable cells. *Sci Rep* 2019;9:9867. 10.1038/s41598-019-46344-z 31285492 PMC6614399

[31] Wells JM, Wilson PW, Le Page RW. Improved cloning vectors and transformation procedure for Lactococcus lactis. *J Appl Bacteriology* 1993;74:629–36. 10.1111/j.1365-2672.1993.tb05195.x 8349525

[32] Junkins EN, Stevenson BS. Using plate-wash PCR and high-throughput sequencing to measure cultivated diversity for natural product discovery efforts. *Front Microbiol* 2021;12. 10.3389/fmicb.2021.675798 PMC8329497 34354680

[33] Momeni B, Brileya KA, Fields MW et al. Strong inter-population cooperation leads to partner intermixing in microbial communities. *eLife* 2013;2:e00230. 10.7554/eLife.00230 23359860 PMC3552619

[34] Liu W, Russel J, Burmølle M et al. Micro-scale intermixing: a requisite for stable and synergistic co-establishment in a four-species biofilm. *ISME J* 1940;12:2018. 10.1038/s41396-018-0112-2 PMC6052071 29670216

[35] van Tatenhove-Pel RJ, de Groot DH, Bisseswar AS et al. Population dynamics of microbial cross-feeding are determined by co-localization probabilities and cooperation-independent cheater growth. *ISME J* 2021;15:3050–61. 10.1038/s41396-021-00986-y 33953364 PMC8443577

[36] Eigentler L, Kalamara M, Ball G et al. Founder cell configuration drives competitive outcome within colony biofilms. *ISME J* 2022;16:1512–22. 10.1038/s41396-022-01198-8 35121821 PMC9122948

[37] Xu S, Van Dyken JD. Microbial expansion-collision dynamics promote cooperation and coexistence on surfaces. *Evolution* 2018;72:153–69. 10.1111/evo.13393 29134631

[38] Bruger EL, Snyder DJ, Cooper VS et al. Quorum sensing provides a molecular mechanism for evolution to tune and maintain investment in cooperation. *ISME J* 2021;15:1236–47. 10.1038/s41396-020-00847-0 33342998 PMC8115533

[39] Zeng X, Zou Y, Zheng J et al. Quorum sensing-mediated microbial interactions: mechanisms, applications, challenges and perspectives. *Microbiol Res* 2023;273:127414. 10.1016/j.micres.2023.127414 37236065

[40] Wu S, Xue Y, Yang S et al. Combinational quorum sensing devices for dynamic control in cross-feeding cocultivation. *Metab Eng* 2021;67:186–97. 10.1016/j.ymben.2021.07.002 34229080

[41] Quispe Haro JJ, Chen F, Los R et al. Optogenetic control of bacterial cell-cell adhesion dynamics: unraveling the influence on biofilm architecture and functionality. *Adv Sci* 2024;11:2310079. 10.1002/advs.202310079 PMC11187914 38613837

[42] Bachmann H, Molenaar D, Kleerebezem M et al. High local substrate availability stabilizes a cooperative trait. *ISME J* 2011;5:929–32. 10.1038/ismej.2010.179 21151005 PMC3105769

[43] Laureys D, Leroy F, Hauffman T et al. The type and concentration of inoculum and substrate as well as the presence of oxygen impact the water kefir fermentation process. *Front Microbiol* 2021;12. 10.3389/fmicb.2021.628599 PMC7904701 33643256

[44] Michielsen S, Vercelli GT, Cordero OX et al. Spatially structured microbial consortia and their role in food fermentations. *Curr Opin Biotechnol* 2024;87:103102. 10.1016/j.copbio.2024.103102 38461750

[45] Tarazanova M, Huppertz T, Beerthuyzen M et al. Cell surface properties of Lactococcus lactis reveal milk protein binding specifically evolved in dairy isolates. *Front Microbiol* 2017;8. 10.3389/fmicb.2017.01691 PMC5594101 28936202

[46] Maeda S, Gasson MJ. Cloning, expression and location of the streptococcus lactis gene for phospho-beta-D-galactosidase. *J Gen Microbiol* 1986;132:331–40. 10.1099/00221287-132-2-331 3086494

