# Supplementary material for: Time of first contact determines cooperator success in a three-member microbial consortium
Source: ISME Commun. 2025 Jan 13;5(1):ycaf004. doi: 10.1093/ismeco/ycaf004 (PMC11879117; doi:10.1093/ismeco/ycaf004)
Supplement: SupplementsRevised_ycaf004 [file supplementsrevised_ycaf004.pdf]

Supplemental Information for "Time of first contact determines cooperator success in a three-member microbial consortium"

Rachel Los[1], Tobias Fecker[2], P.A.M. van Touw[1],
Rinke J. van Tatenhove-Pel[2], Timon Idema[1]

November 21, 2024

1 Department of Bionanoscience, Kavli Institue of Nanoscience, Delft University of Technology, The Netherlands.
2 Department of Biotechnology, Delft University of Technology, The Netherlands.

S1 Supplemental methods

Individual-based simulation details

Each particle is modelled as a spherocylinder that consists of two spheres of diameter, D, connected by a spring with a rest length L (Fig. 1a). This rest length is incrementally increased with growth rate μ until it reaches the division length L_d. The particle then splits up into two daughter particles. Here, a tiny bit of noise is added in the orientation to make sure that the particles don't grow in a perfect line. For each particle pair that is within interaction range, R_{int}, of each other, the repulsive or attractive force is determined. First, the shortest distance between the particles is determined. If it is negative, i.e. there is overlap between the particles or the surface, the repulsive force is calculated as a spring force with spring constant k_r, scaling with the amount of overlap d,

$$F(d) = -k_r \cdot d. \tag{S1}$$

This force is then distributed along the backbone inversely proportional to where along the backbone the overlap occurred.

Particles also experience an attractive force between each other and with the surface. The magnitude of the particle adhesion force depends on the distance r between the centres of the particles and their relative orientations in the following way:

$$F(r,\theta,\phi) = F_{PP} \cdot \left(4\left(\frac{0.95D}{r}\right)^5 - 4\left(\frac{0.95D}{r}\right)^9\right) \cdot \cos\theta \cdot \cos\phi, \qquad \text{for } r > D, \tag{S2}$$

where D is the diameter of the particles, F_{PP} determines the amplitude of the attractive forces, and θ and ϕ are the angles between the backbones of the two particles. The resulting force is then distributed over the two end-points of the particle inversely related to where the closest point between the particles is situated along the length of the particle.

The attractive force to the surface is implemented similarly, but r is now the distance from the center of the particle to the surface.

$$F(r,\theta) = F_{PS} \cdot \left(4\left(\frac{0.95R}{r}\right)^5 - 4\left(\frac{0.95R}{r}\right)^9\right) \cdot \cos\theta, \qquad \text{for } r > R, \tag{S3}$$

where R is the radius of the particles or half the diameter, and θ is the angle the particle makes with the surface. Again the force is distributed over the two spheres that make up the particle, inversely proportional to the distance of each end point to the surface. Consequently, the particles experience both a force and a torque due to the attractive and repulsive interactions.

Every time step all the forces that the particles undergo are calculated and they are moved accordingly. We grow all the particles once every 5 time steps. We grow the system to a set amount of particles and then count how many of those particles are cheaters. Values for all the parameters used can be found in Table S1.

Parameter	Explanation	Value
k_{int}	spring constant of the internal spring	$0.1\ \mathrm{N\,m^{-1}}$
D	particle diameter	1 µm
L_d	length of the spring at division	4 µm
k_r	spring constant of the overlap potential	$0.2\ \mathrm{N\,m^{-1}}$
F_{PS}	particle-surface adhesion prefactor	10^{-5} N
F_{PP}	particle-particle adhesion prefactor	10^{-5} N
DT	division time for a particle growing with growth rate μ	10^5 ts
R_{int}	interaction range for which mechanical interactions are calculated	$2 \cdot L_d$
μ	background growth rate for a cheater particle	$0.0002\cdot\ \mu\mathrm{m}(5 \cdot \mathrm{ts})^{-1}$
K_s	Hill coefficient for the growth rate adjustment	2.5

Table S1: Explanation and values used for the simulation parameters.

Well-mixed model of costly cooperation

In order to show the effect of spatial structure on our simulated consortium, we compare it to a well-mixed system. For this we solve the following system of equations for the evolution of populations of interacting species A, B and C:

$$\frac{dA}{dt} = (1 - c + H(B, N, b))\, A, \tag{S4}$$

$$\frac{dB}{dt} = (1 - c + H(A, N, b))\, B, \tag{S5}$$

$$\frac{dC}{dt} = (1 + H(\min(A, B), N, b))\, C. \tag{S6}$$

(S7)

Here A, B, and C are the size of the population of the corresponding strain. N is the total size of the population. The growth of the populations depends on c, which is the cost of cooperation, and H, which calculates the benefit depending on the ratio of the cooperative species as follows:

$$H(n, N, b) = \frac{\frac{n}{N} \cdot \bar{n}}{K_s + \frac{n}{N} \cdot \bar{n}} \cdot b, \tag{S8}$$

(S9)

where n is the amount of beneficial particles in the total population and N is the size of the total population. Additionally, b is the benefit of cooperation, K_s is the Hill coefficient which is set to 2.5, and $\bar{n}$ is the average number of neighbours a particle has, which is set to 7.5.

The initial conditions are the same as for the simulations, so 10 of each strain, the cheater fraction is measured when a population size of 10^4 particles is reached. The results are summarised in Fig. S2.

A:B:C ratios and how they relate to effective distance between cooperators

If we look from the perspective of a B particle on a plate, the amount of particles that we find within radius, r, away from that particle follows a Poisson distribution,

$$P(k) = \frac{\lambda^k \cdot e^{-\lambda}}{k!}, \text{ with } \lambda = \pi r^2 \sigma. \tag{S10}$$

Here, k is the amount of particles and λ is the expectation value of the amount of particles in a circle of radius r, based on the density of the particles σ. Because we are interested in what the first encounter will be, we want to compare the radii for the first encounter with an A particle and the one with a C particle. The probability of having at least one A particle within a radius, r, is given by,

$$\begin{aligned} P(A) &= 1 - P(0), \\ &= 1 - e^{-\pi r^2 \sigma_A}. \end{aligned} \tag{S11}$$

From this we can get a probability density function,

$$\begin{aligned} f(r) &= \frac{\mathrm{d}}{\mathrm{d}r} P(A), \\ &= 2\pi r \sigma_A \cdot e^{\pi r^2 \sigma_A}, \end{aligned} \tag{S12}$$

which we can then use to find an expectation value of what the distance is that a B will encounter its first neighbour of type A.

$$\begin{aligned} \langle r_{AB} \rangle &= \int_0^\infty r \cdot 2\pi r \sigma_A \cdot e^{\pi r^2 \sigma_A} \, \mathrm{d}r \\ &= \frac{1}{2\sqrt{\sigma_A}}. \end{aligned} \tag{S13}$$

Similarly, the first neighbour of type C is expected to be found at,

$$\langle r_{CB} \rangle = \frac{1}{2\sqrt{\sigma_C}}. \tag{S14}$$

When we subtract the two we get the expected relative distance which denotes who is expected to be further away from B, A or C:

$$\langle r_{AB} - r_{CB} \rangle = \frac{1}{2\sqrt{\sigma_A}} - \frac{1}{2\sqrt{\sigma_C}}. \tag{S15}$$

The particle density at inoculation, σ, can be easily calculated, as it is always the same, namely $3 \cdot 10^6$ cells on a petri dish with a diameter of 9 cm. This gives us an overall initial particle density of

$$\sigma = \frac{3 \cdot 10^6}{\pi (4.5 \cdot 10^4)^2}, \tag{S16}$$

$$= 0.00465 \ \mu\mathrm{m}^{-2} \tag{S17}$$

The densities of A and C are then simply the fractions of those particles times this overall density. $\langle r_{AB} - r_{CB} \rangle$ is plotted for all [C]/[A] ratios used in the 10% cheater fraction experiments in Fig. 4b.

**Strain**	**Carbon source**	**R^2 rep. 1**	**R^2 rep. 2**	**μ_{max} (1/h) ± SD**
MG1363-GFP (Lac^+)	0.09 wt% glc	0.997	0.998	0.67 ± 0.00
MG1363-GFP	0.09 wt% glc	0.997	0.998	0.57 ± 0.01
MG1363-GFP (Lac^+)	0.09 wt% lac	0.996	0.999	0.66 ± 0.00
MG5267	0.09 wt% lac	0.998	0.996	0.64 ± 0.00

Table S2: Goodness-of-fit of all extracted growth curves as determined by the R^2 of the $\ln(OD_{660})$ over time and resulting μ_{max} ± SD (n=2)

Growth study

Fig.S5 shows a representative example of how we calculated the growth rate μ_{max}. As example, we describe the growth study of MG1363-GFP Lac^+ on 0.09 wt% glucose. We measured the OD_{660} from duplicate shake-flasks hourly (Fig. SS5a). We extracted the exponential phase by plotting the log-linear of OD_{660} versus time and fitting a linear curve to the segment where the $\log(OD_{660})$ increased linearly, as determined by the least-squares method (Fig. SS5b). We determined the goodness-of-fit for all replicate curves by R^2 (Table SS2).

Gating strategy

Fig. S6 shows the flow cytometry gating strategy for a representative example.

Step 1: Exclude noise We determined the location of the background noise by the buffer by plotting the forward and side scatter of a sample consisting of only the buffer. This yielded gate 1. From a sample with cells, gate 2 containing everything except the noise was drawn.

Step 2: Determining the fraction of the fluorescent cheater cells Gate 2, containing the cells, was the reference for plotting fluorescence against forward scatter. In this plot, the fluorescent cheater cells were marked as Gate 3. To calculate the cheater fraction, we divided the number of these fluorescent cells by the total cell count, excluding the noise.

S2 Supplemental figures

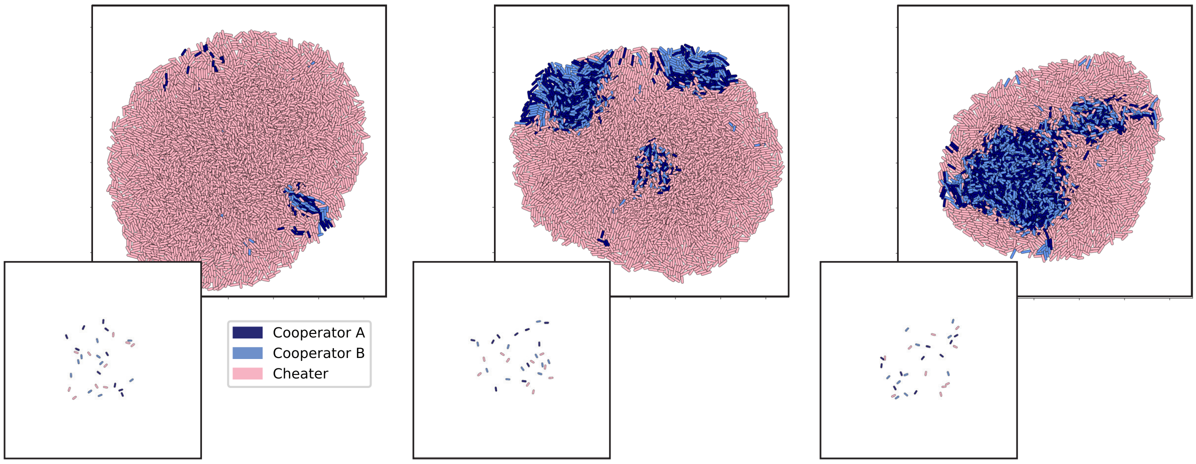

Figure S1: Examples of three different final colonies for the same input parameters for the cross-feeding interaction; value for cost = 0.8 and benefit = 4.0. In the bottom-left corner of each final colony, the initial configuration of that run is shown. Area shown is always 130 × 130 µm^2.

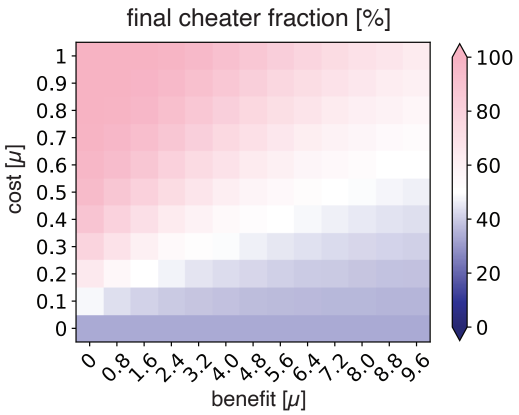

Figure S2: Final cheater fraction for a well-mixed system of A, B and C growing to a population size of 10^4. Interactions are implemented similarly to the IBM, see SI.

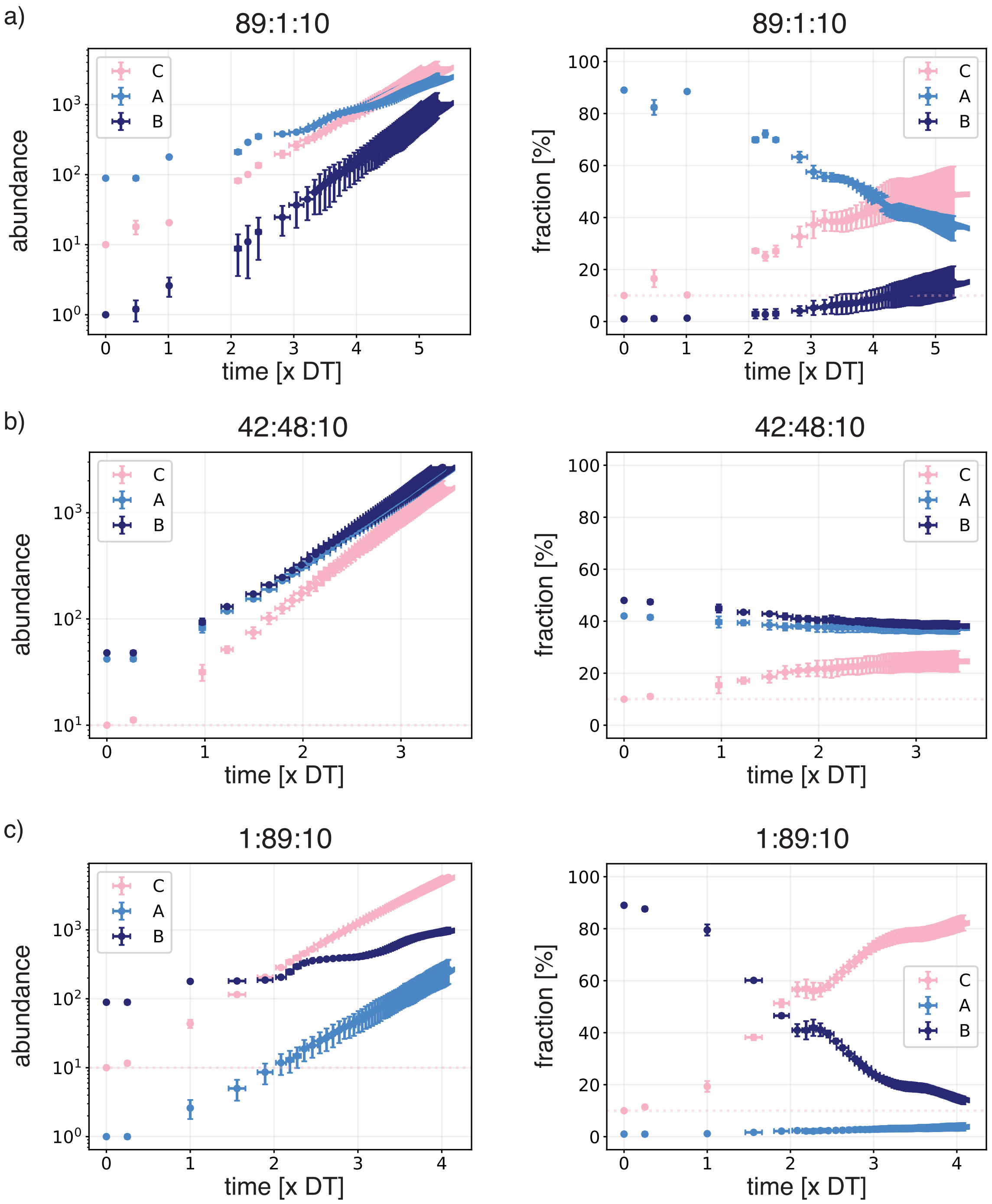

Figure S3: Abundance and fraction of the different particle types over simulation time for the simulation data points of Fig.5b A:B:C ratios a) 89:1:10, b) 42:48:10, and c) 1:89:10. All points are averages and standard deviations of 5 runs.

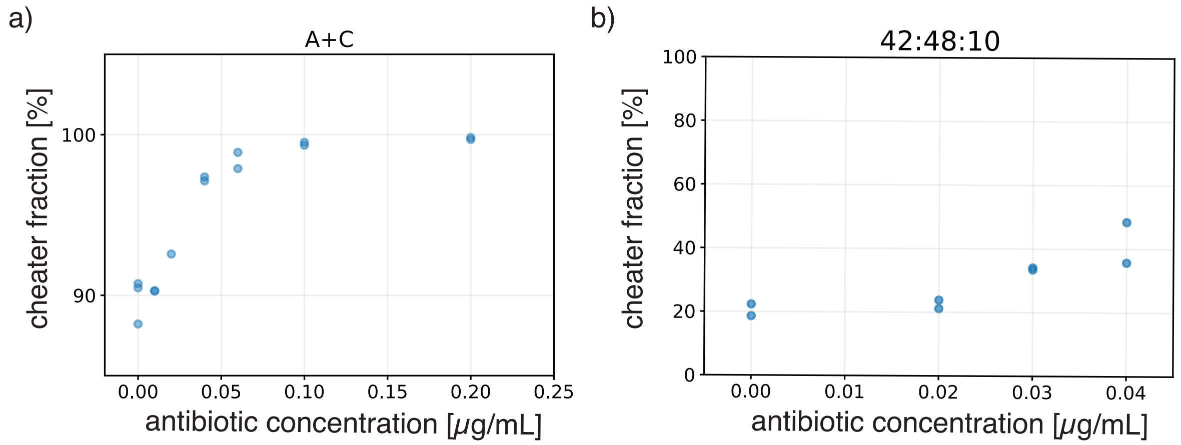

Figure S4: Final cheater fraction for different concentrations of erythromycin a) in plates containing casein and amino acids inoculated with A & C together, and b) in plates containing casein inoculated with A, B & C in a 42:48:10 ratio.

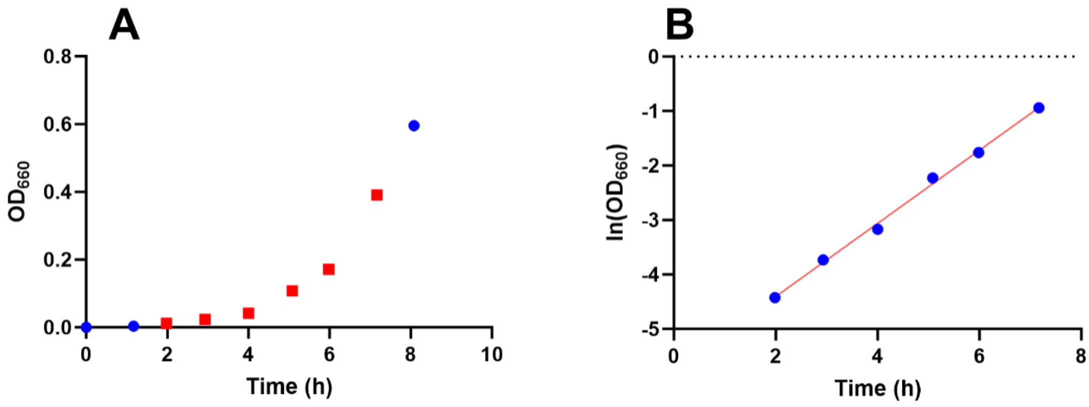

Figure S5: Growth curve of MG1363-GFP Lac^{+} (cheater). a) OD_{660} over time. The data points used to calculate the exponential phase are marked with red squares. b) $\ln(OD_{660})$ of the exponential phase measurements over time. A linear curve was fit to the measurements by least-squares fit.

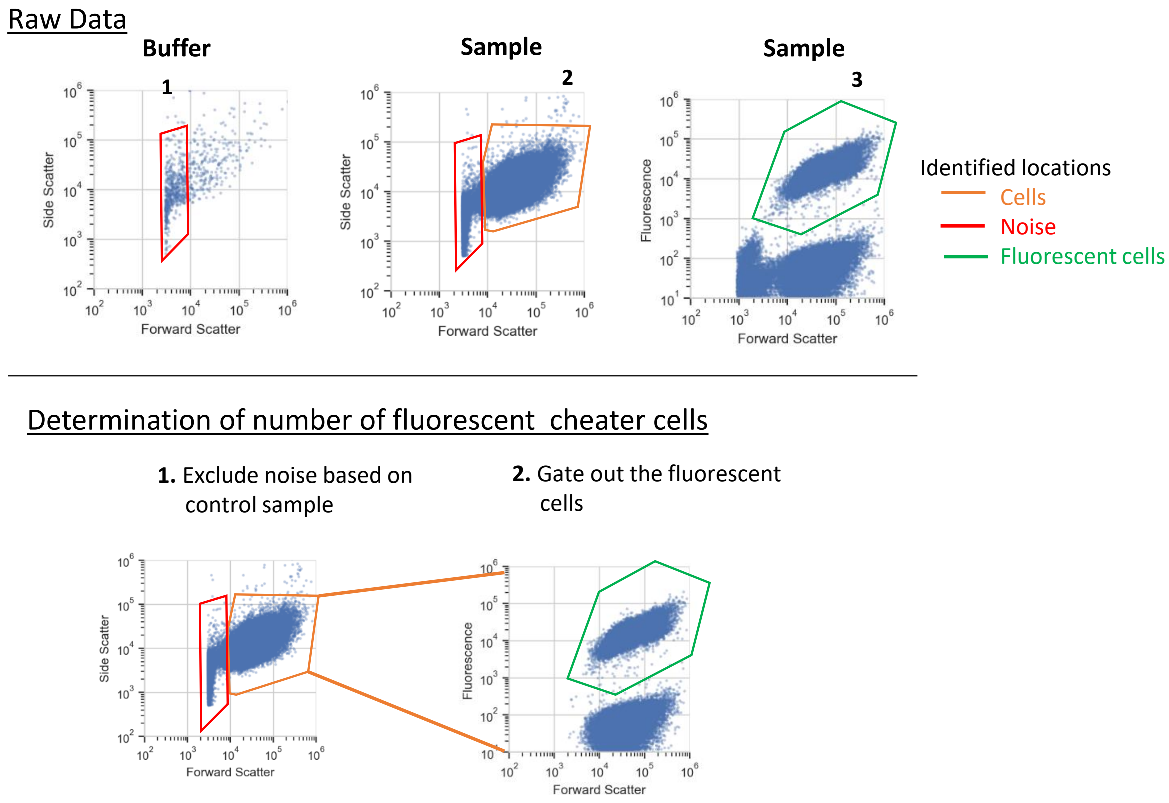

Figure S6: Flow cytometry gating strategy. Areas within the forward scatter – side scatter and fluorescence – forward scatter were identified using control samples. The gating excluded noise and was able to clearly distinguish between fluorescent and non-fluorescent cells to calculate the %cheater fraction.
